# Development and Psychometric Evaluation of the Arabic Version of the Motor Fitness Scale in Saudi Older Adults: A Cross-Cultural Validation Study

**DOI:** 10.3390/healthcare14131887

**Published:** 2026-06-28

**Authors:** Saad M. Alsaad, Juwan Al Musma, Mansour I. Alrasheed, Osama Abdulqader, Ahmed K. Bayoumy, Nasser M. AbuDujain

**Affiliations:** 1 Department of Family and Community Medicine, College of Medicine, King Saud University, Riyadh 11495, Saudi Arabia; 2 Prince Faisal Bin Bandar Chair for Geriatric Research, College of Medicine, King Saud University, Riyadh 11495, Saudi Arabia; 3 College of Medicine, King Saud University, Riyadh 11495, Saudi Arabia; 4 Department of Family Medicine & Polyclinics, King Faisal Specialist Hospital and Research Centre, Riyadh 11211, Saudi Arabia; 5 Tawuniya Insurance Company (Headquarters), Riyadh 13315, Saudi Arabia; 6 Gloucestershire Hospitals NHS Foundation Trust, Gloucester GL53 7AN, UK

**Keywords:** motor fitness, geriatric assessment, scale validation, psychometrics, Arabic translation, functional performance, older adults, Saudi Arabia

## Abstract

**Background and aim:** Motor fitness is a key determinant of functional independence and healthy aging in older adults. The Motor Fitness Scale (MFS) is a simple and widely used instrument for assessing mobility, strength, and balance; however, no validated Arabic version exists. This study aimed to translate, cross-culturally adapt, and evaluate the psychometric properties of the Arabic MFS in a Saudi geriatric population. **Methods:** This cross-sectional validation study was conducted at King Saud University Medical City (2025–2026) among adults aged ≥50 years. Structural validity was examined using confirmatory factor analysis (CFA) with a hierarchical three-factor model (mobility, strength, balance). Reliability was assessed using Cronbach’s alpha, composite reliability, and intraclass correlation coefficient (ICC). Discriminative validity was examined using logistic regression and ROC analysis. **Results:** A total of 140 participants (median age 60 years) were included. CFA supported the second-order three-factor model with good model fit (CFI = 0.986, TLI = 0.983, RMSEA = 0.038). Composite reliability ranged from 0.703 to 0.840 across subscales, and internal consistency was good (α = 0.842). Test–retest reliability was strong (r = 0.797; ICC = 0.831), with no systematic score differences over time. The MFS demonstrated moderate discriminative ability for physical activity status (AUC = 0.671), and higher MFS scores independently predicted physical activity (OR = 1.19, *p* = 0.007). **Conclusions:** The Arabic Motor Fitness Scale demonstrates good structural validity, internal consistency, and test–retest reliability among older adults in Saudi Arabia. The Ar-MFS is a practical and psychometrically sound instrument for assessing motor fitness and functional performance in Arabic-speaking geriatric populations.

## 1. Introduction

As the world’s population ages, it has become more crucial than ever to keep an eye on and support the functional health of the elderly. Motor fitness, which incorporates muscle strength, balance, flexibility, coordination, and mobility, is a vital component of senior health [1]. These qualities are essential for preserving older individuals’ freedom and standard of living. Motor fitness is related to physical activity, although they actually represent different constructs. Motor fitness is a functional capacity measure that includes fundamental physical qualities (strength, balance, mobility), representing the body’s current ability to perform regardless of current activity levels. On the other hand, physical activity refers to patterns of bodily movement over time at various intensities. Although physical activity may support motor fitness, activity alone does not necessarily lead to adequate motor capacity. In contrast, motor fitness can be retained despite a lower level of recent physical activity. Such a conceptual distinction is important when interpreting evidence of construct validity [2].

Based on the above observations, consistent instruments for evaluating senior motor fitness are crucial for directing clinical practice, policy choices, and medical therapies [3]. One such tool, the Motor Fitness Scale (MFS), was developed and validated in Japan and offers an easy, useful, and trustworthy method for evaluating motor fitness across a variety of functional domains in older persons [4]. The MFS is becoming more and more popular in geriatric evaluations around the world, although its use in the older population of Saudi Arabia is yet unknown.

Like many other countries, Saudi Arabia is undergoing a demographic transition characterized by an aging population due to improved healthcare and longer life expectancy. In 2020, people aged 60 and older accounted for around 5.9% of the population, according to the General Authority for Statistics, and this share is expected to increase dramatically over the next several decades [5]. This demographic shift highlights the need for efficient methods to detect functional decline, fall risk, and loss of independence in older persons. There are several functional evaluation instruments available, but many are either too time-consuming, too complicated, or not linguistically or culturally appropriate for the Saudi context. As long as its psychometric properties are confirmed in this particular cultural and linguistic setting, the MFS, which consists of simple observational tasks and scoring criteria, has potential for use in routine geriatric evaluations in Saudi Arabia.

The MFS has demonstrated strong validity and reliability across a variety of settings worldwide. Studies conducted in China and Japan, for example, have shown that MFS scores exhibit high internal consistency and a substantial association with recognized measures of physical function, including grip strength, gait speed, and balance tests [4,6]. These results suggest that the MFS can effectively capture key aspects of motor fitness in older populations. Additionally, it has been applied in community and clinical settings to monitor functional decline, evaluate the effectiveness of interventions, and test for frailty [3]. However, few studies have assessed the MFS’s application beyond East Asian populations, raising questions about how well the results may generalize to other languages and cultural groups.

Physical performance decline varies notably between men and women. Women often experience physiological changes during the menopausal transition. Previous studies have shown greater decline in muscle strength among postmenopausal women compared with premenopausal and perimenopausal women [7]. These changes may be linked to estrogen deficiency, as well as to a reduction in physical activity levels [8]. A study in rodents has shown that estradiol replacement can reverse these effects [9]. It has also been associated with improved bone health and a lower risk of osteoporosis among postmenopausal women, emphasizing the major role of estrogen in maintaining physical activity and muscle function [10].

There remains a dearth of research on standardized motor fitness evaluation instruments for the elderly in the Middle East, particularly in Saudi Arabia. Studies on the health of Saudi Arabia’s older population have mostly concentrated on the prevalence of chronic diseases, cognitive decline, and overall quality of life, with little attention paid to functional measures like motor fitness [11,12]. Tools like the Timed Up and Go (TUG) test and the Short Physical Performance Battery (SPPB) have been used in some local research; however, these tests frequently require specialized equipment or training, which can be a barrier in primary care or rural settings [13,14]. On the other hand, if properly validated, the MFS provides a low-cost, time-efficient, and simple alternative [15].

It is evidently necessary to evaluate whether the MFS retains its validity and reliability in this population, given the distinct sociocultural and environmental factors that may affect motor fitness in older Saudis, such as gender norms, physical activity patterns, and prevalent medical conditions. In geriatric care, a translated and culturally appropriate version of the MFS may be a useful tool for early identification of functional decline and for guiding customized treatment regimens. Thus, this study aimed to translate and culturally adapt the Motor Fitness Scale into Arabic and to examine its content validity, structural validity, internal consistency, test–retest reliability, and construct validity among Saudi older adults.

### Research Purpose and Specific Questions

The present study aimed to translate, culturally adapt, and validate the Arabic version of the Motor Fitness Scale (MFS) among older adults in Saudi Arabia. Establishing the validity and reliability of the Arabic MFS is essential before its implementation in clinical practice and research settings within Arabic-speaking populations. Specifically, this study sought to address the following research questions:

1. Does the Arabic version of the MFS retain the underlying factor structure proposed in the original instrument?
2. Does the Arabic MFS demonstrate adequate internal consistency and test–retest reliability among Saudi older adults?
3. Does the Arabic MFS demonstrate evidence of construct validity through its relationship with established measures of physical activity?
4. Can the Arabic MFS discriminate between physically active and inactive older adults?

## 2. Methodology

### 2.1. Study Design, Setting, and Recruitment

This study employed a cross-sectional methodological design to translate, culturally adapt, and evaluate the psychometric properties of the Arabic version of the Motor Fitness Scale (MFS). The study was conducted among older adults attending geriatric and family medicine clinics at King Saud University Medical City (KSUMC) in Riyadh, Saudi Arabia. Data collection was carried out between Nov 2025 and Feb 2026 using SurveyMonkey (www.surveymonkey.com). The study followed internationally accepted guidelines for cross-cultural adaptation of health measurement instruments and the COSMIN (COnsensus-based Standards for the selection of health Measurement INstruments) recommendations for evaluating measurement properties [16].

Participants were eligible for inclusion if they were aged 50 years or older, Arabic-speaking, able to communicate verbally and provide informed consent, and capable of participating in functional assessment procedures. Participants were excluded if they had severe cognitive impairment preventing reliable communication, had an acute illness or unstable medical condition, or had a severe physical disability that prevented completion of the assessment procedures.

Notably, the inclusion criterion of age ≥ 50 years was used to broaden the applicability of the Arabic version of MFS, allowing early-older adults who might benefit from motor fitness screening in primary care to be included. Such a criterion might also enable us to efficiently compare study findings with other studies investigating early functional decline. Although the original MFS was developed and validated in an older population (adults ≥ 65 years), lowering the age threshold by including the 50–64 age group can identify at-risk individuals who may benefit from preventive interventions. This would facilitate clinical applicability in both primary care and geriatric settings, particularly given the increased focus on early detection of motor deficits in Saudi Arabia.

Following non-probability convenience sampling, participants were recruited from a family medicine center registry of university staff and their dependents. For psychometric validation studies, a commonly recommended sample size is at least 5–10 participants per item, with a minimum of 100 participants for factor analysis [16]. Since the Motor Fitness Scale contains 14 items, a sample size of 100–150 participants was targeted to ensure adequate power for reliability and validity analyses. For the test–retest reliability analysis, a subsample of approximately 26 participants was invited to complete the retest assessment within 7–14 days of the initial administration, an interval commonly recommended for test–retest reliability studies to balance recall bias and true change in the measured construct.

### 2.2. Questionnaire and Scales

The data collection instrument consisted of four sections. The first section collected demographic and functional information, including age, gender, marital status, functional mobility (independent walking, use of walking aids, or wheelchair use), and income, to describe the study population and their functional characteristics. The second section included the Arabic Motor Fitness Scale (MFS), which assessed motor fitness and functional performance, with emphasis on balance, mobility, and overall physical function in older adults. The third and fourth sections comprised additional functional assessments used to evaluate construct validity, including the International Physical Activity Questionnaire (IPAQ), which evaluates the frequency and duration of physical activity across different intensity levels, and the Physical Activity Scale for the Elderly (PASE), which measures physical activity specifically among older adults.

Eligible participants were recruited during their clinic visits. After obtaining informed consent, trained research assistants administered the questionnaires and functional assessments, including the Arabic Motor Fitness Scale, IPAQ, and PASE. These instruments were administered during the same assessment session in accordance with standardized procedures (Supplementary Figure S1).

#### 2.2.1. Motor Fitness Scale

The Motor Fitness Scale (MFS) is a self-administered questionnaire developed in 1998 by T. Kinugasa and H. Nagasaki for community-dwelling older adults. It consists of 14 items evaluating three domains: mobility, strength, and balance. It includes six items to assess mobility, including the ability to climb stairs, walk without breathlessness, jump, run short distances, pass another walker, and complete a 30-min nonstop walk. Also, four items assess strength as the ability to carry a 10-pound object, lift a 20-pound object, pick up a fallen bicycle, and open a screw-type bottle cap. Additionally, there are 4 items for balance evaluation: touching the floor with fingertips and extended knees, dressing while standing without support, standing up from a chair without using the hands, and standing on the toes without support. Responses were recorded as “Yes,” which scored as 1, or “No,” which scored as 0. By summing all answers, a final score ranging from 0 to 14 was obtained, with higher scores indicating better motor fitness. The Motor Fitness scale showed excellent internal consistency (α = 0.92) and high test–retest reliability. Also demonstrated strong validity for assessing functional limitations and predicting disability in older adults, not only those with severe functional limitations [4].

#### 2.2.2. International Physical Activity Questionnaire (IPAQ) Short Form

The International Physical Activity Questionnaire (IPAQ) was developed in 1998 by the International Consensus Group to provide a standardized, well-validated instrument for estimating physical activity across populations. It is available in two versions, the 31-item long form (IPAQ-LF) and the 9-item short form (IPAQ-SF). The short form scores physical activity across four intensity levels: vigorous-intensity activity, moderate-intensity activity, walking, and sitting. The authors recommend using the last 7-day IPAQ-SF recall as it reduces participant burden and provides more accurate self-reporting. It showed high reliability (α < 0.80) and good test–retest stability. Also, it offers acceptable validity for assessing physical activity habits, particularly frequency and duration; validity varies depending on which index or objective measure is used as the standard [17]. We used the Arabic version of the IPAQ-SF, which also shows moderate-to-good test–retest reliability (ICC = 0.66 to 0.96), supporting its use in our study [18,19].

#### 2.2.3. The Physical Activity Scale for the Elderly (PASE)

The Physical Activity Scale for the Elderly (PASE) was created in 1993 by Dr. James A. Washburn. It is an easy, self-reported instrument for assessing physical activity over the last 7 days in individuals aged 65 years or older by evaluating three main domains: occupational, household, and leisure activities. The score is based on the frequency, duration, and intensity of activity, yielding a final result ranging from 0 to 793, with higher scores indicating better physical activity. PASE showed high reliability and acceptable test–retest reliability with a reported test–retest reliability coefficient (0.75). It also showed good validity compared to objective measures of physical activity and energy expenditure, making it an accurate indicator of physical activity in the elderly [20,21].

### 2.3. Translation and Cultural Adaptation

A standardized forward–backward translation protocol was followed after obtaining the necessary permission from the tool’s publisher [22,23]. Two professional translators, fluent in Arabic and English (one with medical expertise), independently translated the MFS from English into Arabic. These versions were reviewed and merged into a single draft during consensus meetings with S.M.A. and N.M.A.

We then pilot-tested this draft among 27 elderly participants to ensure the clarity and comprehensibility of the language and its cultural appropriateness. Minor refinements were made based on the participants’ feedback. Afterward, the tool was back-translated into English by a blinded, independent, bilingual expert. Two native English speakers reviewed the back-translated version, confirming its semantic and content accuracy. Once these stages were finalized, the Ar-MFS was formally set as the instrument for the subsequent psychometric analysis.

### 2.4. Ethical Consideration and Compliance

The study was approved by the Institutional Review Board (IRB) at King Saud University (E-25-10037), ensuring that study procedures were conducted in accordance with the ethical principles outlined in the Declaration of Helsinki. Participation was voluntary, and participants were informed that they could withdraw at any time and that their privacy and anonymity would be maintained. Permission to use the Motor Fitness Scale (MFS) was obtained under license from Springer Nature (License Number: 6011470648244), 17 April 2025.

### 2.5. Theoretical Positioning: Motor Fitness, Functional Performance, and Physical Activity

The MFS was designed to evaluate motor fitness, a multidimensional functional capacity construct representing an individual’s ability to perform activities requiring physical strength, balance, and mobility. Motor fitness is an independent entity from physical activity (which refers to the most recent behavioral movement patterns, including frequency, duration, and intensity). Although these constructs are theoretically linked (for example, physical activity might maintain fitness), they measure different dimensions of health. MFS is an assessment of functional capacity, while IPAQ and PASE are instruments designed to assess behavioral activity levels. Therefore, a weak correlation between capacity and behavior is expected and theoretically coherent, since an individual can maintain adequate motor fitness levels despite lower recent physical activity.

### 2.6. Statistical Analysis and Software

The validity of the MFS was examined using confirmatory factor analysis (CFA) within a structural equation modeling framework. Given the dichotomous nature of MFS items, the weighted least squares mean and variance adjusted (WLSMV) estimator with theta parameterization was applied. In line with the original MFS structure, a hierarchical (second-order) model was tested. This higher-order structure was selected because motor fitness is theoretically conceptualized as a single construct comprising three interrelated domains (mobility, strength, balance) rather than independent factors. The approach allows for both the estimation of the general Motor Fitness construct and its relevant dimensions, in agreement with the multidimensionality of functional capacity characteristics in older adults.

Three first-order latent factors were specified: Mobility, Strength, and Balance. Motor Fitness was modeled as a second-order latent factor to account for the shared variance among the three first-order dimensions. Model fit was evaluated using multiple goodness-of-fit indices, including the scaled chi-square statistic, Comparative Fit Index (CFI), Tucker–Lewis Index (TLI), Root Mean Square Error of Approximation (RMSEA) with 90% confidence interval, and Standardized Root Mean Square Residual (SRMR). Acceptable model fit was determined based on conventional criteria (CFI and TLI ≥ 0.90–0.95, RMSEA ≤ 0.08 with preference ≤ 0.05, and SRMR ≤ 0.08–0.10). Composite reliability (ω) for each first-order factor was calculated using SEM-based reliability estimates. Internal consistency of the MFS was further assessed using Cronbach’s alpha. Spearman’s rank correlation coefficient was used to assess test–retest reliability (the correlation between baseline and retest MFS scores), as well as construct validity (the correlations between MFS total scores and IPAQ and PASE scores). Agreement between test and retest total scores was additionally examined using a two-way mixed-effects intraclass correlation coefficient (ICC) with absolute agreement. The difference between baseline and retest total scores was assessed using the paired Wilcoxon signed-rank test. Item-level stability was evaluated by calculating both percent agreement and Cohen’s kappa for each dichotomous MFS item between baseline and retest responses. To examine discriminative validity, logistic regression analysis was conducted to assess whether MFS scores predicted physical activity status (physically active vs. inactive). The model included MFS score as the primary predictor, with adjustment for demographic variables (age, gender, nationality, monthly income, marital status) and clinical characteristics (comorbid conditions).

Multicollinearity was assessed using variance inflation factors (VIF). Model assumptions were evaluated by examining influential observations using standardized residuals. Model discrimination was further evaluated using receiver operating characteristic (ROC) curve analysis, and the area under the curve (AUC) was calculated. Missing data were handled using listwise deletion for each analysis, with sample sizes reported for each outcome. Due to incomplete data collection, IPAQ and PASE were administered to subsamples only (IPAQ: n = 40, 71.4% missing; PASE: n = 62, 55.7% missing). All statistical analyses were conducted using RStudio (version 2024.9.1.394, Boston, MA, USA) using R version 4.4.2. Confirmatory factor analysis was conducted using the lavaan package; reliability analyses were conducted using the irr and psych packages; and logistic regression and ROC analysis were performed using base R functions. The level of statistical significance was set at *p* < 0.05.

## 3. Results

### 3.1. Demographic and Functional Characteristics of Patients

Initially, we received 147 responses on the online data collection platform. However, seven patient records with missing MFS responses were excluded. Therefore, a total of 140 participants were included in the study. The median age was 60.0 years (IQR: 55.0–65.0). More than half of the participants were male (56.4%), and the majority were Saudi nationals (90.0%). Most participants were married (89.3%). Regarding monthly income, the largest proportion reported earning 5000–14,999 SAR (34.3%). The majority of participants walked independently (92.9%), with fewer using a cane (5.7%) or a wheelchair (1.4%). Nearly half of the participants (48.6%) reported being physically active for more than 6 months, while 15.7% had been active for less than 6 months; smaller proportions reported intending to initiate physical activity within the next 30 days (17.9%) or 6 months (12.9%), and 5.0% reported no intention to start within the next 6 months. In general, physically active respondents accounted for 64.3% of the sample. Most participants (84.3%) reported having one or more comorbid conditions (Table 1).

### 3.2. Confirmatory Factor Analysis

The second-order three-factor model demonstrated good overall fit to the data: χ^2^(75) = 89.96, *p* = 0.115; CFI = 0.986; TLI = 0.983; RMSEA = 0.038; SRMR = 0.133. The non-significant chi-square test and low RMSEA indicated an adequate fit to the observed covariance matrix, while the incremental fit indices exceeded the recommended thresholds. Although the SRMR value of 0.133 was slightly greater than traditional thresholds (≤0.08–0.10), suggesting low residual misfit, this is not uncommon for dichotomous items and is outweighed by the overall strong performance of the incremental fit indices (CFI, TLI) and a theoretically consistent factor structure.

**Figure 1:**
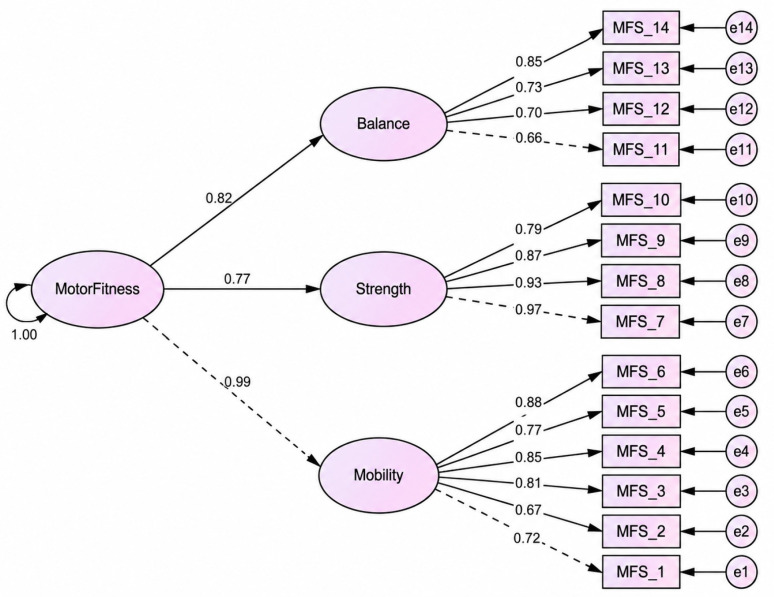
Confirmatory factor analysis of the Arabic Motor Fitness Scale (MFS). The model depicts a second-order Motor Fitness construct underlying three first-order latent domains (Balance, Strength, and Mobility). Standardized factor loadings are shown for all item–factor and factor–construct relationships. Rectangles denote observed variables and circles denote latent variables.

As presented in Figure 1, all first-order standardized factor loadings were substantial. Loadings ranged from 0.671 to 0.881 for the mobility subscale, 0.794 to 0.973 for the strength subscale, and 0.661 to 0.852 for the balance subscale, indicating strong associations between the observed indicators and their respective latent constructs. At the second-order level, the general Motor Fitness factor loaded strongly on the three subscales (Figure 1). Standardized second-order factor loadings were: Mobility λ = 0.991 (95% CI: 0.979–0.999), Strength λ = 0.767 (95% CI: 0.628–0.906), and Balance λ = 0.817 (95% CI: 0.697–0.937). All three CIs are narrow and precise, indicating stable estimates and exceptionally strong relationships. Composite reliability coefficients supported internal consistency: ω = 0.804 for Mobility, ω = 0.840 for Strength, and ω = 0.703 for Balance, all meeting or approaching acceptable reliability thresholds.

### 3.3. Reliability and Stability Analysis

The correlation between the second-order latent factor score and the total raw MFS score was very high (r = 0.964, 95% CI: 0.951–0.974, *p* < 0.001), indicating that approximately 93% of the variance in the total score was explained by the underlying second-order Motor Fitness construct (r^2^ = 0.93). Internal consistency reliability of the MFS was good (Cronbach’s alpha = 0.842, 95%CI: 0.792 to 0.877) for the 14-item scale.

**Figure 2:**
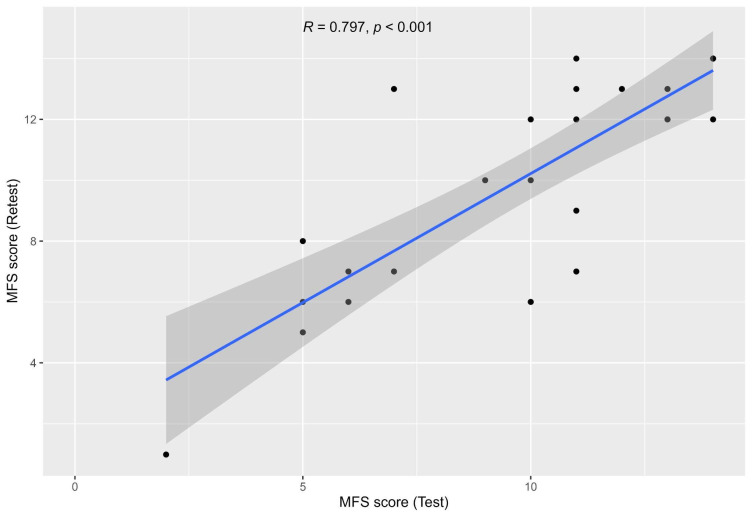
A scatterplot showing the correlation between test and retest MFS scores.

**Figure 3:**
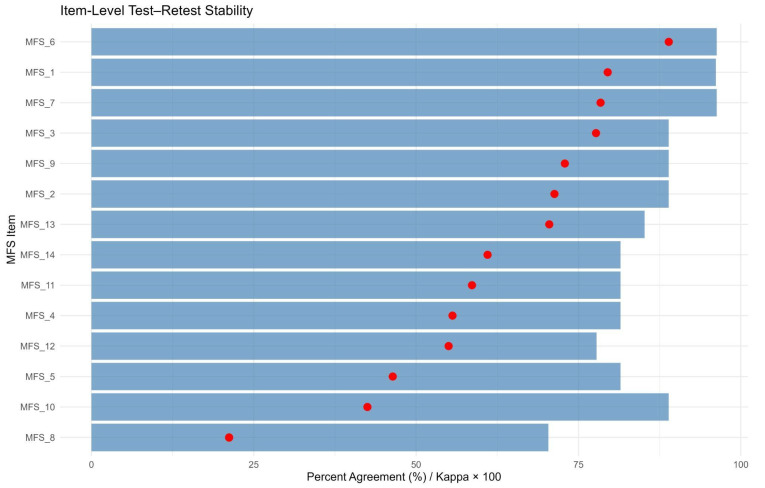
Percent agreement (bars) and Cohen’s kappa coefficients (red dots) between baseline and retest responses for each Motor Fitness Scale item.

Test–retest reliability was assessed in a subsample of 26 participants with available scores in a test–retest dataset. A strong and statistically significant correlation was observed between baseline and retest total scores (r = 0.797, *p* < 0.001, Figure 2). No significant difference was found between baseline and retest scores using the Wilcoxon signed-rank test (V = 65.5, *p* = 0.614), indicating that scores were consistent over time. At the item level, percent agreement between baseline and retest ranged from 70.4% to 96.3%, with most items demonstrating agreement above 80% (Figure 3). ICC analysis showed good reliability for the total score (ICC(A,1) = 0.831, 95% CI: 0.659–0.920, *p* < 0.001). Individual item Cohen’s kappa coefficients for MFS items ranged from κ = 0.212 (MFS_8) to κ = 0.889 (MFS_6), with a median of 0.711. This suggests that most individual MFS items have substantial agreement. The low kappa for MFS_8 (κ = 0.212) may be due to the small test–retest subsample (n = 26), which increases variability in item-specific estimates.

### 3.4. Description of the Scores of the Domains Under Study

The median MFS score at baseline (n = 140) was 11.0 (IQR: 8.0–13.0). At time 2 (n = 26), the median MFS score was 12.0 (IQR: 7.0–13.0). The median IPAQ and PASE scores were 1695.0 (IQR: 585.0–3546.0) and 109.8 (IQR: 59.6–151.4), respectively (Table 2). However, IPAQ and PASE responses were based on patient subgroups with available records (n = 40 and n = 62, respectively).

### 3.5. Convergent Validity

**Figure 4:**
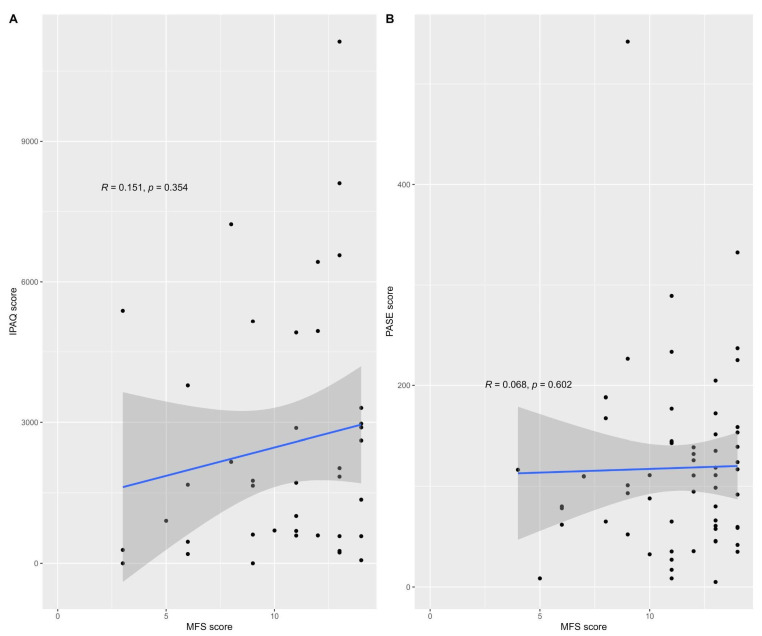
Convergent validity analysis of the Arabic Motor Fitness Scale (Ar-MFS). Scatterplots showing the relationships between total Ar-MFS scores and physical activity measures. (**A**) IPAQ score (r = 0.151, *p* = 0.354, n = 40). (**B**) PASE score (r = 0.068, *p* = 0.602, n = 62). Each point represents one participant.

Convergent validity analysis showed weak, non-significant correlations between MFS and IPAQ (r = 0.151, *p* = 0.354) and PASE (r = 0.068, *p* = 0.602, Figure 4). However, to assess whether missingness in the IPAQ and PASE data had influenced the results, we conducted a post hoc power analysis, which indicated insufficient power to detect small-to-moderate correlations (power = 15% for IPAQ and 8% for PASE). These outcomes suggest that the findings should be interpreted cautiously, as the IPAQ and PASE analyses were underpowered to detect small correlations (there is a risk of false negatives).

### 3.6. Discriminative Validity (Predictors of Being Physically Active)

**Figure 5:**
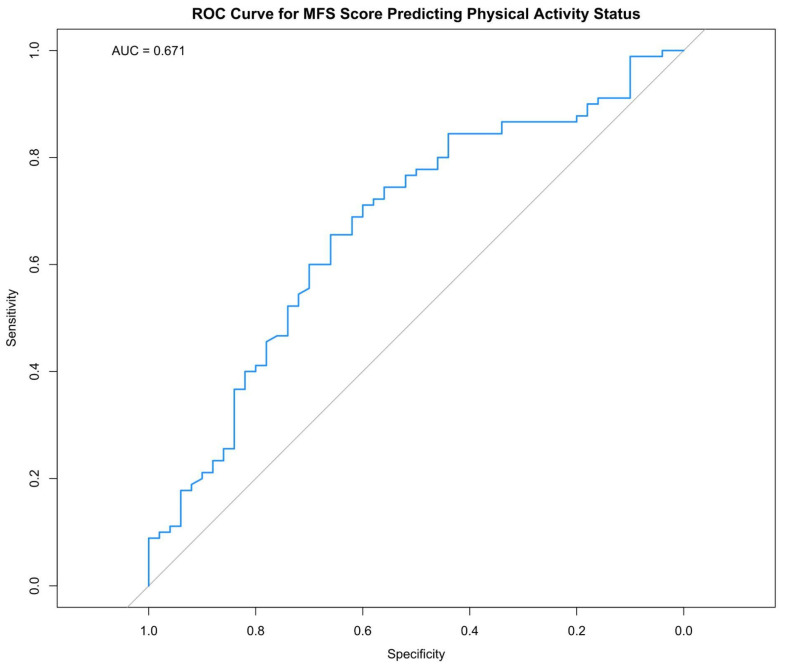
Receiver operating characteristic (ROC) curve of the logistic regression model predicting physical activity status.

Multicollinearity was assessed using the VIF, and all predictors showed VIF values close to 1, indicating no evidence of multicollinearity. No influential observations had standardized residuals greater than ±3, indicating the absence of influential outliers. The ROC curve analysis demonstrated that the MFS score had an area under the curve (AUC) of 0.671 for predicting physical activity status (Figure 5). Although the MFS score showed moderate ability to discriminate between physically active and inactive participants, the observed AUC reflected only limited discriminatory performance. The optimal MFS cutoff score for predicting physical activity was 0.59 (sensitivity = 76.7%, specificity = 60.0%), based on the Youden index.

Overall, the model assumptions were satisfied in the regression analysis. Specifically, the Hosmer–Lemeshow goodness-of-fit test indicated adequate model fit (*p* = 0.435), McFadden’s R^2^ = 0.084; multicollinearity was absent (all VIF < 5); and no influential observations were identified (all Cook’s distances < 1). The regression analysis showed that only the MFS score was significantly associated with physical activity. Higher MFS scores were associated with increased odds of physical activity (OR = 1.19, 95% CI, 1.05 to 1.36, *p* = 0.007) after adjustment for demographic and clinical characteristics. No significant associations were observed for age, gender, nationality, monthly income, comorbid conditions, or marital status (all *p* > 0.05, Table 3).

## 4. Discussion

The present study aimed to translate, culturally adapt, and psychometrically validate the Motor Fitness Scale for use among Arabic-speaking older adults in Saudi Arabia. To our knowledge, this is the first validation of the MFS in Arabic. Overall, the findings are encouraging and support the structural integrity, reliability, and practical applicability of the Arabic version. The confirmatory factor analysis replicated the hierarchical structure proposed in the original development study, with Mobility, Strength, and Balance loading onto a second-order Motor Fitness construct. Model fit indices were strong across multiple parameters, suggesting that the theoretical structure of motor fitness underpinning the original instrument is preserved in this population. The use of a higher-order model supports the interpretation of motor fitness as a global construct composed of several interrelated functional domains. In practical terms, the second-order factor represents overall motor fitness, whereas the first three factors provide complementary information about specific aspects of physical functioning. The Mobility domain reflects locomotor capacity and endurance, the Strength domain captures functional muscular performance, and the Balance domain represents postural control and flexibility [3]. In addition, internal consistency was good, and test–retest analyses demonstrated stability over time.

With regard to structural validity, our findings are consistent with the original work by Kinugasa and Nagasaki, who demonstrated a higher-order structure composed of three interpretable subdomains [4]. In the original cohort of community-dwelling Japanese older adults, the scale showed excellent internal consistency (α = 0.92) and strong test–retest reliability (0.92), alongside meaningful associations with summary physical performance scores [4]. In the present study, standardized factor loadings across all domains were substantial, and composite reliability coefficients ranged from acceptable to good. Although Cronbach’s alpha (0.842) was lower than that reported in the original study, it remains well within acceptable thresholds for multidimensional instruments and likely reflects contextual and demographic differences rather than structural weakness. Our sample included adults aged 55 years and older, with a high prevalence of comorbid conditions (84.3% with one or more), which may introduce greater heterogeneity in functional performance than in the original Japanese sample aged 65 years and above [4].

Convergent validity analyses showed very weak positive correlations between the MFS and both the International Physical Activity Questionnaire (IPAQ-SF) and the Physical Activity Scale for the Elderly (PASE). These associations were non-significant and should be interpreted with caution, particularly given the limited subsample sizes and a post hoc power analysis indicating insufficient power to detect small-to-moderate correlations. However, beyond statistical considerations, the magnitude of correlation is conceptually coherent. The MFS assesses motor fitness as a functional capacity construct encompassing balance, muscular strength, and mobility. In contrast, IPAQ and PASE quantify recent physical activity behavior in terms of frequency, duration, and intensity across various domains [20,24]. Although physical activity helps maintain motor fitness, the constructs are not interchangeable. Previous research has demonstrated that motor fitness reflects underlying physical capacity rather than behavioral activity patterns, reinforcing the conceptual distinction between functional fitness assessments and self-reported physical activity measures [3]. An individual may report high levels of activity that are predominantly low-intensity or non-strengthening, which may not translate into optimal balance or strength performance. Conversely, an older adult may maintain relatively preserved motor capacity despite reporting lower recent activity levels. Therefore, the weak correlations observed support the theoretical distinction between behavioral physical activity and functional motor fitness, rather than undermining construct validity.

While higher MFS scores independently predicted physical activity (OR = 1.19, *p* = 0.007), discriminative validity was modest (AUC = 0.671). This indicates that MFS had a limited capacity to classify individuals as active or inactive in terms of performing physical activity. Such findings highlight that the MFS, which was primarily developed to determine functional capacity rather than as a behavioral activity screening tool, has a limited ability to distinguish activity status.

Discriminative validity analyses provide additional support for the clinical relevance of the Arabic MFS. The scale demonstrated a moderate ability to discriminate between physically active and inactive individuals (AUC = 0.671), and higher MFS scores independently predicted physical activity after adjustment for demographic and clinical covariates. The absence of multicollinearity and influential observations strengthens confidence in this model. Although the discriminative capacity is not high, it is reasonable given that the MFS was not designed as a behavioral activity screening tool. Instead, it captures functional capability. Evidence from longitudinal studies indicates that physical fitness indicators are closely linked with functional outcomes and may predict future decline in daily living abilities among older adults [25]. The finding that motor fitness is independently associated with physical activity status reinforces the scale’s practical relevance while still reflecting the conceptual separation between the two constructs.

Reliability and reproducibility analyses further support the stability of the Arabic MFS. Internal consistency was good (α = 0.842), and composite reliability estimates across subscales were acceptable. Test–retest reliability demonstrated strong temporal stability, with a significant correlation between baseline and follow-up scores (r = 0.797) and a good intraclass correlation coefficient (ICC = 0.831). Importantly, there was no evidence of systematic change between time points, as indicated by the non-significant Wilcoxon signed-rank test. Item-level agreement was high, with percent agreement exceeding 80% in most cases. While the retest subsample was relatively small, the consistency of correlation-based and agreement-based metrics suggests that the instrument yields reproducible results over short-term intervals.

The higher-order model structure has implications for clinical interpretation. The second-order motor fitness scoring yielded a robust functional capacity index that might be utilized for population screening and monitoring. At the same time, each of the three subscales (Mobility, Strength, Balance) enables more precise evaluation within specific functional areas to guide targeted interventions. The total score can be used by clinicians for risk stratification, while subscale patterns can be used to identify domain-specific deficits that require targeted rehabilitation. This suggested framework highlights the clinical utility of the Arabic version of MFS as a potential diagnostic tool and a comprehensive assessment instrument for identifying functional deficits among Arabic-speaking older adults.

Several limitations should be acknowledged. First, the study was conducted in a single tertiary academic center in an urban setting. This might have limited the external applicability of the findings to rural and underserved regions with different levels of healthcare access, physical environments and social determinants of health. Therefore, although participants represented a range of functional abilities, generalisability to rural populations or other Arabic-speaking countries may be limited. Additionally, we used a screening age inclusion criterion of ≥50 years. Although appropriate for early- to older-adult primary care screening purposes, the age threshold applies to a different demographic than the original MFS validation (≥65 years) or traditional geriatric cutoffs. This could have limited direct comparison with previous validation studies. Another limitation is that physical activity was assessed using self-reported measures rather than objective tools, which may have introduced reporting bias [26]. Future research should examine the predictive validity of the Arabic MFS for forecasting falls, hospitalization, disability progression, and general frailty. This direction is particularly relevant given growing evidence that objective measures of physical fitness can serve as early indicators of functional decline and loss of independence in aging populations [25]. Multicentre studies across different Arabic-speaking regions would strengthen external validity. Finally, substantial data missingness in IPAQ and PASE was not anticipated during study design. This pattern of missingness has limited testing of convergent validity. Post hoc power analysis indicated insufficient power to detect correlations, making the weak findings difficult to interpret. Future studies should prospectively collect data on convergent measures to adequately assess relationships between motor fitness and physical activity behavior. Despite these limitations, the current findings provide a necessary foundation for integrating structured motor fitness assessment into geriatric and elderly care practice in Saudi Arabia and potentially across the wider Middle East.

## 5. Conclusions

Our Arabic version of the MFS demonstrates a reliability profile comparable to that of the original instrument and maintains its underlying theoretical structure. These findings support its use as a practical, low-burden tool for assessing motor fitness in Arabic-speaking geriatric populations. In clinical settings where time and resources may be limited, the Arabic MFS offers a structured yet simple approach to identifying early functional decline and monitoring motor fitness over time.

## Figures and Tables

**Table 1:** Demographic and functional characteristics of patients.

Characteristic	Description
Age	60.0 (55.0–65.0)
Gender	
Male	79 (56.4%)
Female	61 (43.6%)
Nationality	
Saudi	126 (90.0%)
Non-Saudi	14 (10.0%)
Marital status	
Single	5 (3.6%)
Married	125 (89.3%)
Divorced	4 (2.9%)
Widowed	6 (4.3%)
Monthly income (SAR)	
<5000	20 (14.3%)
5000 to 14,999	48 (34.3%)
15,000 to 29,999	27 (19.3%)
30,000 or more	11 (7.9%)
Prefer not to disclose	34 (24.3%)
Functional status	
Walking independently without assistance	130 (92.9%)
Walking using a cane	8 (5.7%)
Partially using a wheelchair (can walk short distances)	2 (1.4%)
Physical activity stages of change	
Yes, for more than 6 months	68 (48.6%)
Yes, for less than 6 months	22 (15.7%)
No, but I intend to do it within the next 30 days	25 (17.9%)
No, but I intend to do it within the next 6 months	18 (12.9%)
No, I don’t intend to do it within the next 6 months	7 (5.0%)
Comorbid conditions	
None	22 (15.7%)
One condition or more	118 (84.3%)

Median (Q1–Q3); n (%).

**Table 2:** Description of the scores of the domains under study.

Characteristic	Description
MFS score, n = 140	
Median (Q1–Q3)	11.0 (8.0–13.0)
Mean (SD)	10.3 (3.4)
Min–Max	0.0–14.0
MFS score (time 2), n = 26	
Median (Q1–Q3)	12.0 (7.0–13.0)
Mean (SD)	10.2 (3.6)
Min–Max	1.0–14.0
IPAQ score, n = 40	
Median (Q1–Q3)	1695.0 (585.0–3546.0)
Mean (SD)	2506.3 (2618.0)
Min–Max	0.0–11,128.8
PASE score, n = 62	
Median (Q1–Q3)	109.8 (59.6–151.4)
Mean (SD)	118.1 (88.3)
Min–Max	5.0–542.1

**Table 3:** Predictors of being physically active.

Characteristic	OR	95% CI	*p*-Value
MFS score	1.19	1.05, 1.36	0.007
Age	1.04	0.99, 1.11	0.139
Gender			
Male	Reference	Reference	
Female	2.01	0.79, 5.32	0.149
Nationality			
Saudi	Reference	Reference	
Non-Saudi	0.62	0.18, 2.22	0.449
Monthly income (SAR)			
<5000	Reference	Reference	
5000 to 14,999	0.95	0.27, 3.28	0.934
15,000 to 29,999	1.08	0.25, 4.68	0.915
30,000 or more	1.62	0.24, 14.8	0.637
Prefer not to disclose	0.94	0.25, 3.35	0.919
Comorbid conditions			
None	Reference	Reference	
One condition or more	0.31	0.08, 0.98	0.063
Marital status			
Single/Divorced/Widowed	Reference	Reference	
Married	0.80	0.21, 2.79	0.730

Abbreviations: CI = Confidence Interval; OR = Odds Ratio.

## Data Availability

Data used in this study are available upon reasonable request from the corresponding author.

## Supplementary Materials

The following supporting information can be downloaded at: https://www.mdpi.com/article/10.3390/healthcare14131887/s1, Figure S1: Flow Diagram of Participant Recruitment, Screening, and Inclusion in the Psychometric Validation Study.

## Abbreviations

MFS	Motor Fitness Scale
IPAQ	International Physical Activity Questionnaire
IPAQ-LF	International Physical Activity Questionnaire Long Form
IPAQ-SF	International Physical Activity Questionnaire Short Form
PASE	Physical Activity Scale for the Elderly
CFA	Confirmatory Factor Analysis
SEM	Structural Equation Modeling
WLSMV	Weighted Least Squares Mean and Variance Adjusted estimator
CFI	Comparative Fit Index
TLI	Tucker–Lewis Index
RMSEA	Root Mean Square Error of Approximation
SRMR	Standardized Root Mean Square Residual
ω	Composite Reliability (McDonald’s Omega)
α	Cronbach’s Alpha
ICC	Intraclass Correlation Coefficient
ROC	Receiver Operating Characteristic
AUC	Area Under the Curve
VIF	Variance Inflation Factor
CI	Confidence Interval
IQR	Interquartile Range
OR	Odds Ratio
r	Correlation Coefficient
R^2^	Coefficient of Determination
IRB	Institutional Review Board
SAR	Saudi Riyal
