# Supplementary material for: Development and Psychometric Evaluation of the Arabic Version of the Motor Fitness Scale in Saudi Older Adults: A Cross-Cultural Validation Study
Source: Healthcare (Basel). 2026 Jun 28;14(13):1887. doi: 10.3390/healthcare14131887 (PMC13361477; doi:10.3390/healthcare14131887)
Supplement: Supplementary file 1 [file healthcare-14-01887-s001.zip › Arabic Version of MFS.pdf]

جامعة الملك سعود
King Saud University

مقياس اللّياقة الحركية

القدرة الحركية	نعم (١)	لا (٠)
١. باستطاعتي الصعود إلى الطابق الثاني والنزول منه.		
٢. باستطاعتي الصعود إلى الطابق الثاني دون أن ألهث.		
٣. استطيع القفز في الهواء بحيث تكون كلتا قدمي فوق الارض في نفس الوقت.		
٤. باستطاعتي الركض مسافة عشرين خطوة.		
٥. باستطاعتي أن أتخطى شخصًا آخر يتقدمني أثناء المشي.		
٦. باستطاعتي الاستمرار في المشي أكثر من ٣٠ دقيقة.		
**القوة**	**نعم (١)**	**لا (٠)**
٧. باستطاعتي أن أحمل شيئًا وزنه يقارب ٥ كيلوغرام (مثل عبوة الحليب سعة جالون واحد).		
٨. باستطاعتي أن أرفع شيئًا وزنه يقارب ١٠ كيلوغرام (مثل زجاجتي حليب سعة جالون واحد).		
٩. باستطاعتي أن أرفع درّاجة هوائية سقطت على الأرض.		
١٠. باستطاعتي أن أفتح زجاجة ذات غطاء لولبي.		
**التوازن**	**نعم (١)**	**لا (٠)**
١١. باستطاعتي أن ألمس الأرض بأطراف أصابع يديّ في وضع الوقوف دون أن أثني ركبتيّ.		
١٢. باستطاعتي أن ألبس جوربًا أو بنطالًا أو تنورة أثناء الوقوف دون الحاجة لأن استند بشيء.		

King Saud University

١٣. باستطاعتي الوقوف من وضعية الجلوس دون أن استخدم يديّ.		
١٤. باستطاعتي الوقوف على أطراف أصابع قدميّ دون الحاجة لأن استند بشيء.		

King Saud University

اشتملت الإجابات عن البنود الإجابات البسيطة الآتية:

"نعم" (قادر على القيام بالحركة المذكورة في الوقت الحالي) أو "لا" (غير قادر)، حيث تُساوي كل إجابة بـ "نعم" درجة واحدة وكل إجابة بـ "لا" صفر. إذا كانت الحركة المذكورة في البند غير مألوفة لدى المستجيب، سُئل المستجيب إذا كان يظن أنه قادر على القيام بها أو لا. مقياس اللّياقة الحركية هو مجموع درجات الأربعة عشر بندًا، أو عدد البنود التي أُجيب عنها بـ "نعم"، لضمان أن تعكس الدرجة الكلية (وأعلاها ١٤ درجة) بدقة اللّياقة الحركية لدى المستجيبين.

Do not use without permission. Please ensure proper citation of both the original paper by Kinugasa & Nagasaki. (1998) and the Arabic validation paper by Alsaad *et al.* (2026) when using this version in your article.
