# Supplementary material for: Development and Psychometric Evaluation of the Arabic Version of the Motor Fitness Scale in Saudi Older Adults: A Cross-Cultural Validation Study
Source: Healthcare (Basel). 2026 Jun 28;14(13):1887. doi: 10.3390/healthcare14131887 (PMC13361477; doi:10.3390/healthcare14131887)
Supplement: Supplementary file 1 [file healthcare-14-01887-s001.zip › English Version of MFS.pdf]

Motor Fitness Scale

Mobility

1. I can walk up to and down from the second floor.
2. I can walk up to the second floor without getting out of breath.
3. I can jump up in the air so that both feet are clearly off the floor at the same time.
4. I can run for 20 steps.
5. I can pass another person who is walking ahead of me.
6. I can keep walking for over 30 minutes.

Strength

7. I can carry something weighing 10 pounds (e.g., a 1 gallon milk bottle).
8. I can lift something weighing 20 pounds (e.g., two 1 gallon milk bottles).
9. I can pick up a fallen bicycle.
10. I can open a screw-type bottle cap.

Balance

11. I can touch the floor with my fingertips while standing with extended knees.
12. I can put on a sock, slacks, or a skirt while standing with no support.
13. I can stand up from a chair without using my hands.
14. I can stand on my toes without any support.

Responses to the items consisted of simply:

"yes" (able to perform a specific action at present) or "no" (unable), with a score of 1 for every "yes", and 0 for every "no". If the subjects were not used to doing the action, they were asked whether they thought they could do it or not. The Motor Fitness Scale was the sum total of the 14 items, or the number of items answered with "yes", to ensure that the score (maximum 14 points) would accurately indicate the motor fitness of the subjects.
