# Supplementary figures and images for: Development and Psychometric Evaluation of the Arabic Version of the Motor Fitness Scale in Saudi Older Adults: A Cross-Cultural Validation Study
Source: Healthcare (Basel). 2026 Jun 28;14(13):1887. doi: 10.3390/healthcare14131887 (PMC13361477; doi:10.3390/healthcare14131887)

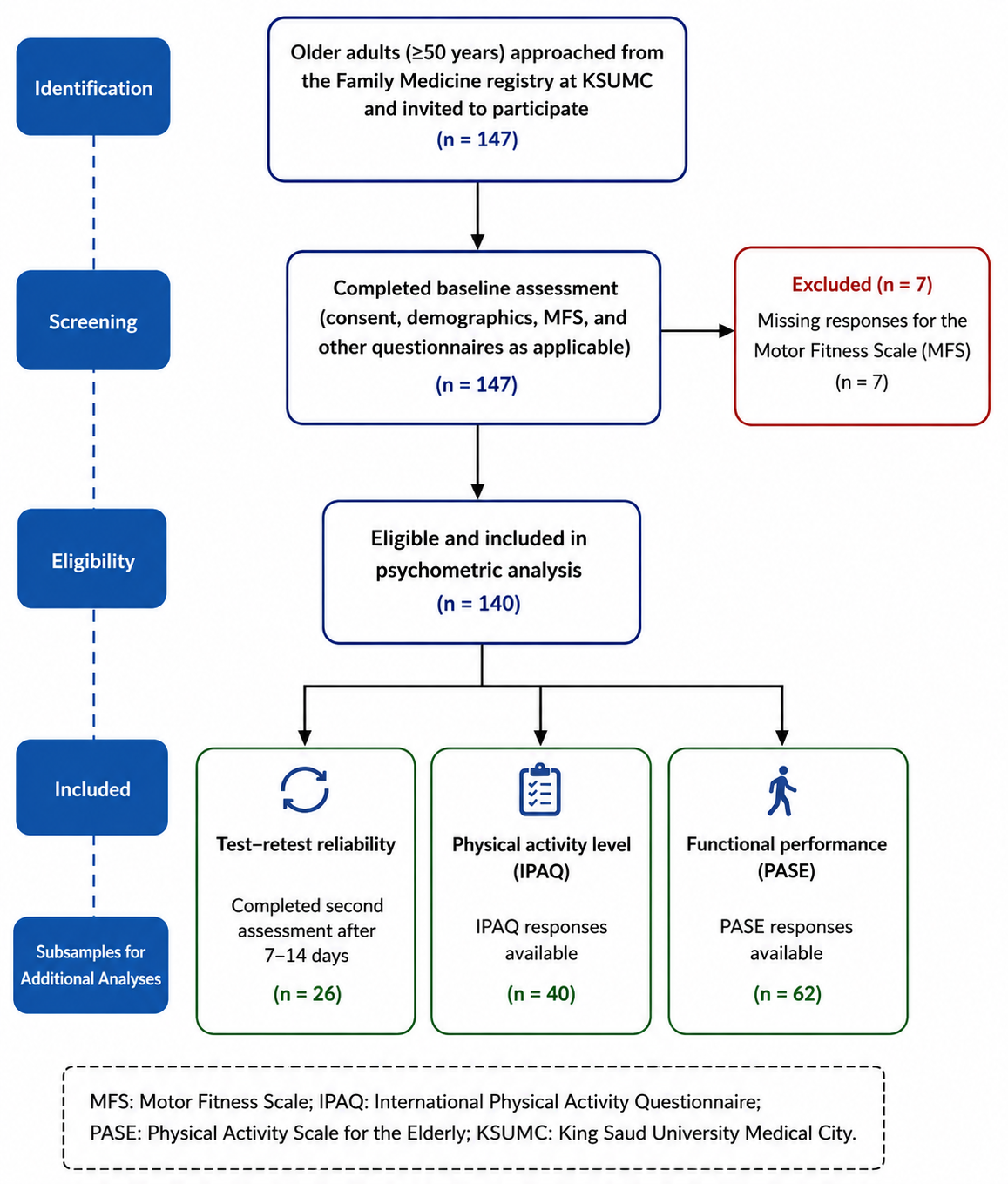

Supplement: Supplementary file 1 [file healthcare-14-01887-s001.zip › Supplementary Figure S1.png]
